# Bone marrow involvement by classic Hodgkin lymphoma posttransplant lymphoproliferative disorder

**DOI:** 10.1002/jha2.258

**Published:** 2021-07-04

**Authors:** Kirill A. Lyapichev, Amanda Calleroz, Andrea N. Marcogliese

**Affiliations:** ^1^ Department of Pathology The University of Texas Medical Branch Galveston Texas USA; ^2^ Department of Hematopathology The University of Texas MD Anderson Cancer Center Houston Texas USA; ^3^ Department of Pathology & Immunology Baylor College of Medicine Houston Texas USA

A 9‐year‐old boy with a history of hypoplastic left heart syndrome status postorthotopic heart transplant at the age of 3 months presented with pancytopenia, low‐grade fever, shortness of breath, and rising Epstein‐Barr virus (EBV) titers.

Decision on performing bone marrow biopsy was made. The specimen showed an atypical infiltrate consisting of rare large, Reed‐Sternberg‐like cells with prominent nucleoli (panel A, smear, hematoxylin and eosin stain [H&E], 1000×). CD20 was weakly positive in these pleomorphic cells (B, CD20, 1000×). PAX5 showed dim nuclear staining in CD30‐positive Reed‐Sternberg‐like cells (C, CD30/PAX5, 1000×). The in situ hybridization (ISH) of EBV‐encoded RNA (EBER) was positive in most of large, pleomorphic cells (D, EBER (ISH), 1000×).


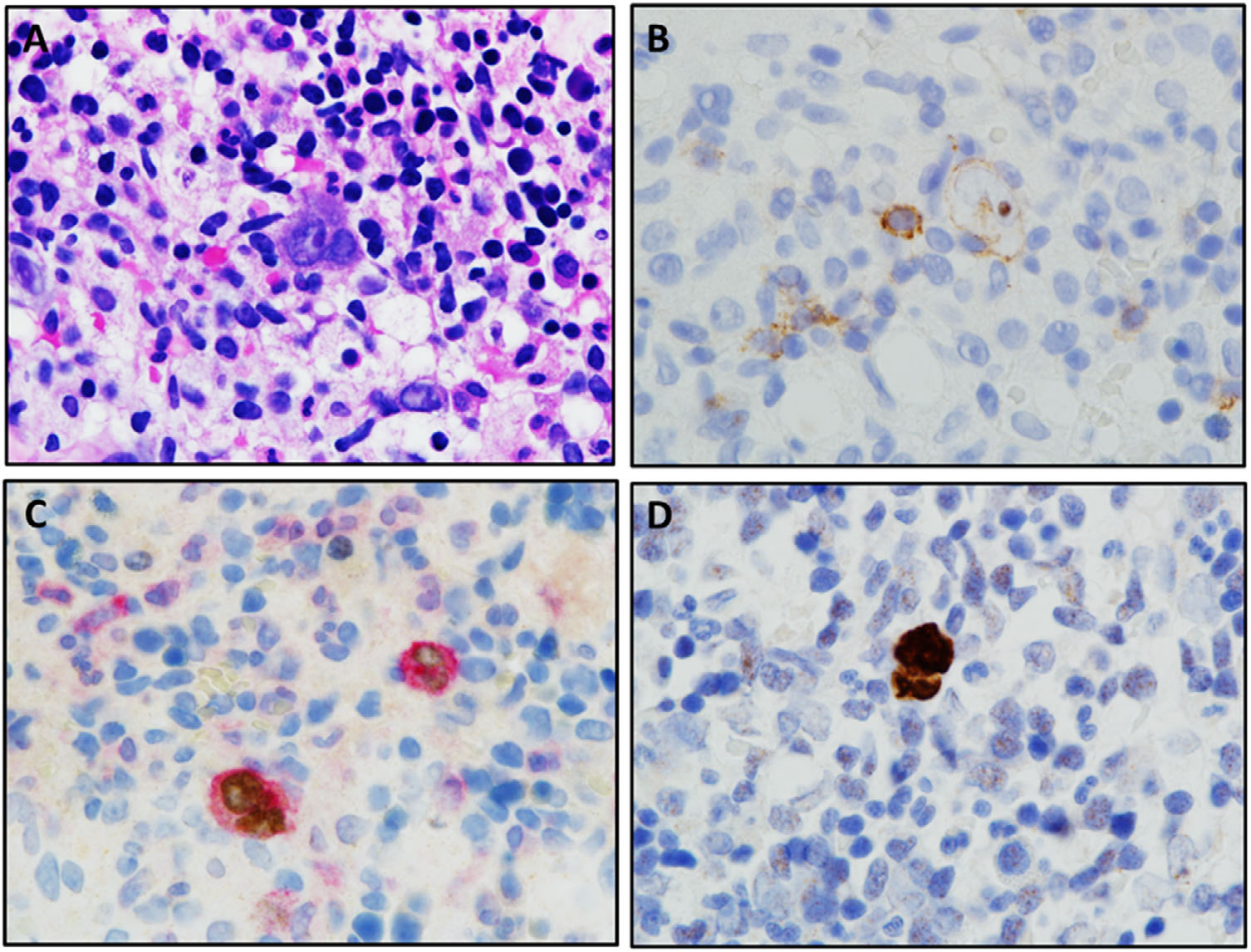


This rare and interesting case represents bone marrow involvement by classic Hodgkin lymphoma (CHL) posttransplant lymphoproliferative disorder. To avoid misdiagnosis, it is important to remember that this type of neoplasm is characteristic for posttransplant patients; the neoplastic cells should be EBER positive and more likely to show B‐cell antigen expression like CD20 than CHL in immunocompetent hosts.

